# Complete Genome Sequence of *Leifsonia xyli* subsp. *cynodontis* Strain DSM46306, a Gram-Positive Bacterial Pathogen of Grasses

**DOI:** 10.1128/genomeA.00915-13

**Published:** 2013-11-07

**Authors:** Claudia Barros Monteiro-Vitorello, Marcelo Marques Zerillo, Marie-Anne Van Sluys, Luis Eduardo Aranha Camargo, João Paulo Kitajima

**Affiliations:** Departamento de Genética, Escola Superior de Agricultura “Luiz de Queiroz,” Universidade de São Paulo, Piracicaba, São Paulo, Brazila; Departamento de Botânica, Instituto de Biociências, Universidade de São Paulo, São Paulo, Brazilb; Departamento de Fitopatologia, Escola Superior de Agricultura “Luiz de Queiroz,” Universidade de São Paulo, Piracicaba, São Paulo, Brazilc; Mendelics Análise Genômica, São Paulo, Brazild

## Abstract

We announce the complete genome sequence of *Leifsonia xyli* subsp. *cynodontis*, a vascular pathogen of Bermuda grass. The species also comprises *Leifsonia xyli* subsp. *xyli*, a sugarcane pathogen. Since these two subspecies have genome sequences available, a comparative analysis will contribute to our understanding of the differences in their biology and host specificity.

## GENOME ANNOUNCEMENT

*Leifsonia xyli* subsp. *cynodontis* is a pathogen that causes stunting in Bermuda grass (*Cynodon dactylon*), and *Leifsonia xyli* subsp. *xyli* is the causal agent of the sugarcane ratoon stunting disease (1). Among other species of *Leifsonia*, *L. xyli* subsp. *cynodontis* and *L. xyli* subsp. *xyli* are the only plant pathogens. Herein, we describe the complete sequencing of the *L. xyli* subsp. *cynodontis* genome using a combination of strategies. Thirteen bacterial artificial chromosome (BAC) inserts and 768 BAC ends were sequenced with Sanger platforms as a pilot project. Illumina HiSeq reads were produced with 100 cycles of paired-end runs, yielding >17.5 million 100-base-long reads for each end. *L. xyli* subsp. *cynodontis* whole DNA was finally submitted to a PacBio RSII, generating 28,645 reads with a total yield of 135,470,327 bp with an average read length of 4,729 bp. Genome assembly was performed by combining Mira (4) v3.4.0.1 and CLC Genomics Workbench v 6.5. Mira contigs served as references for genome finishing, and CLC contigs were used as confirmation of the assembly. All Mira assembly gaps were closed with PacBio reads using a pipeline based on cross_match v1.090518. Illumina reads were then realigned against the final genome sequence using CLC Genomics Workbench in order to correct wrong bases and improve the quality of low-complexity regions. BAC inserts and end sequences were used to confirm the final assembly.

The finishing process produced a genome sequence consisting of 2,686,418 bp, with a G+C content of 68%. The total genome size is compatible with that determined in previous experiments using pulse-field gel electrophoresis (3). GeneMark.hmm for Prokaryotes (version 2.8) (5) automatically generated gene predictions using a precomputed *L. xyli* subsp. *xyli* gene model (*Lxx*_CTCB07). Genome annotation was compared to prediction and function categories assigned for the *L. xyli* subsp. *xyli* genome (2) and automatically transferred to *L. xyli* subsp. *cynodontis* according to the criteria of ≥80% amino acid sequence identity and ≥80% query and subject coverage. Small open reading frames (ORFs) (≤60 amino acids) not meeting the above criteria were manually inspected and deleted if no significant BLASTP match was detected using GenBank NR as the reference database. The genome sequence of *L. xyli* subsp. *cynodontis* contains 2,470 protein-coding genes, 46 tRNA genes, and 1 rRNA operon, and it is 102,260 bp longer than that of *L. xyli* subsp. *xyli*. Currently, 80 pseudogenes have been identified. A total of 627 ORFs were specific to *L. xyli* subsp. *cynodontis*, considering a BLASTP E value of ≤1 × 10^–5^, whereas 750 ORFs were specific to *L. xyli* subsp. *xyli*. According to the *L. xyli* subsp. *xyli* annotation platform, specific ORFs of *L. xyli* subsp. *cynodontis* were assigned to the following categories: (i) 19.6% intermediary metabolism; (ii) 13.2% small molecule biosynthesis; (iii) 11.3% macromolecule metabolism; (iv) 9.4% cell structure; (v) 10.8% cellular processes; (vi) 3% mobile genetic elements; (vii) 4.6% pathogenicity, virulence, and adaptation; (viii) 27.9% conserved or hypothetical proteins; and (ix) 3.3% unable to classify. Highlights among the specific elements of the *L. xyli* subsp. *cynodontis* genome sequence are the genes encoding membrane-associated proteins, hemolysins, hemagglutinins, and transposable elements. Further comparative analysis and lab experiments will improve our understanding of the differential relationships established by the two subspecies with their hosts and of the characteristics of other species of *Leifsonia*.

### Nucleotide sequence accession numbers.

This genome sequence has been deposited at DDBJ/EMBL/GenBank under the accession number CP006734. The version described in this paper is the first version, CP006734.1.
